# Increases in Plasma Lutein through Supplementation Are Correlated with Increases in Physical Activity and Reductions in Sedentary Time in Older Adults

**DOI:** 10.3390/nu6030974

**Published:** 2014-03-03

**Authors:** Rebecca L. Thomson, Alison M. Coates, Peter R. C. Howe, Janet Bryan, Megumi Matsumoto, Jonathan D. Buckley

**Affiliations:** 1 Nutritional Physiology Research Centre, Sansom Institute for Health Research, University of South Australia, Adelaide, South Australia 5001, Australia; E-Mails: alison.coates@unisa.edu.au (A.M.C.); janet.bryan@unisa.edu.au (J.B.); jonathan.buckley@unisa.edu.au (J.D.B.); 2 Clinical Nutrition Research Centre, University of Newcastle, Callaghan, New South Wales 2308, Australia; E-Mail: peter.howe@newcastle.edu.au; 3 Department of Physical Education, College of Humanities and Sciences, Nihon University, Tokyo 156-8550, Japan; E-Mail: matsumoto.megumi@nihon-u.ac.jp

**Keywords:** lutein, carotenoid, physical activity, aging, sedentary, accelerometer

## Abstract

Cross-sectional studies have reported positive relationships between serum lutein concentrations and higher physical activity levels. The purpose of the study was to determine whether increasing plasma lutein levels increases physical activity. Forty-four older adults (BMI, 25.3 ± 2.6 kg/m^2^; age, 68.8 ± 6.4 year) not meeting Australian physical activity guidelines (150 min/week of moderate to vigorous activity) were randomized to consume capsules containing 21 mg of lutein or placebo with 250 mL of full-cream milk per day for 4 weeks and encouraged to increase physical activity. Physical activity was assessed by self-report, pedometry and accelerometry (daily activity counts and sedentary time). Exercise self-efficacy was assessed by questionnaire. Thirty-nine participants competed the study (Lutein = 19, Placebo = 20). Lutein increased plasma lutein concentrations compared with placebo (*p* < 0.001). Absolute and percentage changes in plasma lutein were inversely associated with absolute (*r* = −0.36, *p* = 0.03) and percentage changes (*r* = −0.39, *p* = 0.02) in sedentary time. Percentage change in plasma lutein was positively associated with the percentage change in average daily activity counts (*r* = 0.36, *p* = 0.03). Exercise self-efficacy did not change (*p* = 0.16). Lutein increased plasma lutein, which was associated with increased physical activity and reduced sedentary time in older adults. Larger trials should evaluate whether Lutein can provide health benefits over the longer term.

## 1. Introduction

The populations of many developed countries are ageing. Ageing predisposes to physical and mental disability, but this can be attenuated through regular physical activity. Regular physical activity can maintain physical function during ageing through reductions in the loss of bone tissue [1], maintenance of cartilage [2] and skeletal muscle mass [3], and can also assist in maintaining mental function by improving cognitive performance [4]. However, despite a range of public health initiatives to increase population levels of physical activity, calls for communities to engage more in physical activity have in large part gone unheeded, with only ~40% of people engaging in sufficient physical activity to promote or maintain good health [5]. Clearly, alternative approaches are required to assist people in increasing their levels of physical activity.

Data from cross-sectional studies have demonstrated positive associations between serum levels of the carotenoids lutein and zeaxanthin and physical activity, such that higher serum lutein and zeaxanthin are associated with higher levels of physical activity [6,7]. It has been implied that the higher serum lutein and zeaxanthin are a result of increased physical activity [6], but because these studies are cross-sectional they are unable to determine causality, and it is possible that physical activity levels might be higher due to higher circulating lutein and zeaxanthin concentrations (*i.e*., higher lutein and zeaxanthin might increase physical activity). Such a hypothesis has not previously been tested. 

The aim of this study was to determine whether an increase in plasma lutein concentration from consuming lutein capsules with full-fat milk as a vehicle, increased physical activity in older adults.

## 2. Experimental Section

### 2.1. Participants

**Figure 1:**
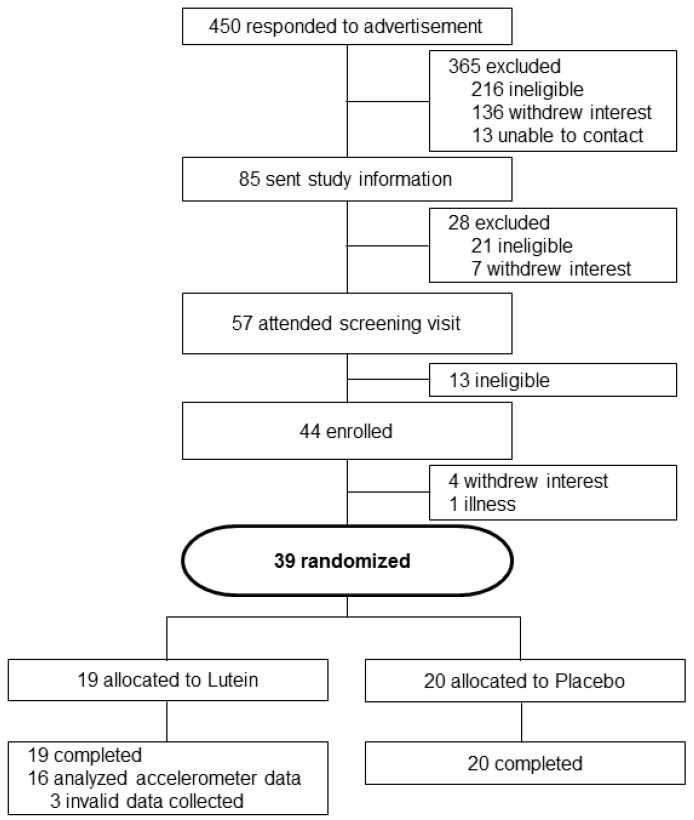
CONSORT Diagram of participant flow from recruitment to analysis of the study.

Forty four older adults (55–80 years) who were not obese (BMI 18.5–29.9 kg/m^2^) and who by self-report did not meet current Australian national physical activity guidelines (*i.e*., not undertaking at least 150 min of moderate to vigorous activity per week) were recruited for the study (Figure 1). Participants were excluded if they were not weight-stable or had changed medication in the last 12 weeks, if they had diabetes, lactose intolerance or allergy to cow’s milk protein, Coeliac disease, an acute or terminal illness, history of smoking in the last 12 months, moderate or severe cognitive impairment, or consumed more than four alcoholic drinks per day. Prior to participation all volunteers were screened by a qualified medical practitioner, and undertook an exercise stress test. They were excluded if they had a medical condition that might be exacerbated by exercise, if exercise might place them at risk, or if they had a condition that might limit exercise training.

### 2.2. Protocol

The study comprised a randomized double-blind placebo-controlled intervention trial of 5 weeks duration. After a 1 week run-in period, participants were allocated to consume 3 capsules per day containing either 7 mg of lutein and also 0.3 mg of zeaxanthin (total of 21 mg/day of lutein and 0.9 mg/day of zeaxanthin; Lutein) or 3 placebo capsules per day (154.5 mg microcrystalline cellulose, 154.5 mg lactose, 4 mg brown cacao and 2 mg safflower colour per capsule; Placebo). The capsules were all similar in appearance and were consumed with 250 mL of full cream milk (3%–4% fat). Full cream milk was used to provide a vehicle to promote better absorption of the fat soluble carotenoids. Allocation to treatment was via minimisation [8], aimed at minimising differences in gender, age, and current levels of physical activity (assessed by pedometry in the week prior to treatment allocation). Participants were asked to consume the milk and capsules at the same time every day, preferably in the morning. After 1 week of supplementation all participants were encouraged to try and achieve at least 150 min/week of moderate to vigorous activity in accordance with the national physical activity guidelines for Australian adults [9]. Participants were also asked to limit their consumption of carotenoid rich foods during the study. Data were collected between May 2011 and May 2012.

**Figure 2:**
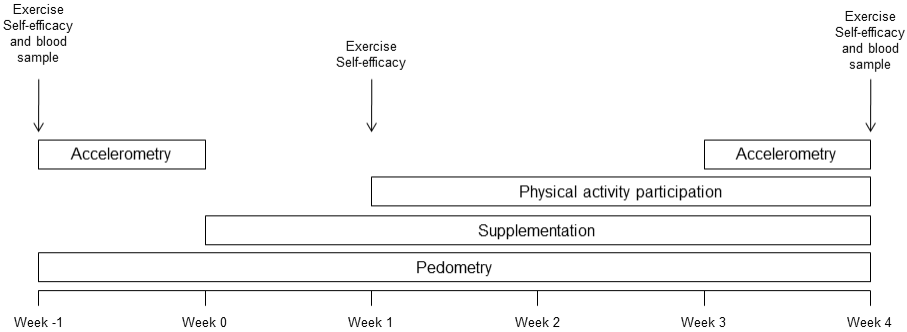
Study design.

Physical activity levels were assessed by pedometry throughout the study period and by accelerometry during the one week run-in prior to commencement of supplementation and during the final week of supplementation. Exercise self-efficacy was assessed by questionnaire [10] at the start of the run-in week (Week −1) and again after one week of supplementation, but prior to participant being encouraged to undertake physical activity, in order to assess any effects of the supplements on exercise self-efficacy without any potential confounding effects of physical activity participation. Participants were then encouraged to commence regular physical activity in accordance with Australian national physical activity guidelines for adults [9]. Physical activity levels were assessed again by accelerometry during the final week and exercise self-efficacy was assessed at the end of the final week. A diagram of the protocol is provided in Figure 2. The trial was approved by the Human Research Ethics Committee of the University of South Australia (21 February 2011) and was registered on the Australia New Zealand Clinical Trials Register (ACTRN12611000248965, 7 March 2011). Written informed consent was obtained from all participants.

### 2.3. Physical Activity Program

Information was provided to participants on the benefits of regular physical activity for maintaining and promoting health and participants were encouraged to achieve 150 min of moderate to vigorous physical activity per week in line with the national physical activity guidelines for Australian adults [9] which promote 30 min of moderate-intensity physical activity on most days, increased incidental activity, and vigorous exercise where possible. Participants were offered advice to achieve these guidelines by undertaking regular walking and/or jogging exercise, but were free to decide how much exercise they did each week.

### 2.4. Outcome Assessments

Participants maintained exercise diaries in which they recorded the duration of exercise undertaken each day during Weeks 1 to 4 to allow for subsequent calculation of self-reported physical activity. Accelerometry (7164 ActiGraph, MTI Health Services, Fort Walton Beach, FL, USA) was used over a 7−day period (5 week days and 2 weekend days) during the run-in and again in the final week of intervention to ascertain the amount of sedentary activity, light physical activity and moderate-vigorous physical activity undertaken. Cut-points for activity levels using the accelerometer data were determined in accordance with definitions applied to accelerometer data in the National Health and Nutrition Examination Survey [11]. Participants were also provided with pedometers and asked to record the number of steps taken each day throughout the study.

Exercise self-efficacy was assessed using a validated questionnaire portraying different levels of exercise task demands against which participants rated the strength of their belief in their ability to perform their exercise routine regularly using a 100 point scale [10]. The higher the score the greater the exercise-self efficacy.

Fasting blood samples were taken at the first visit (before supplementation) and at the end of the study. Samples were frozen at −80 °C until subsequent analysis in October 2012. Plasma lutein and zeaxanthin concentrations in these samples were measured by High Performance Liquid Chromatography using the method of Jensen *et al*. [12]. Total cholesterol was measured using an enzymatic method using a Seimens Advia 2400 analyser.

### 2.5. Statistical Analysis

Statistical analysis was performed using IBM SPSS Statistics 21 (SPSS, Chicago, IL, USA) and statistical significance was set at *p* < 0.05. Data were checked for normality prior to analysis and are presented as mean ± standard deviation (SD). Baseline measurements were compared between groups using independent samples *t*-tests. Correlation analysis using Pearson’s correlation coefficient was used to determine relationships between the changes in plasma lutein and zeaxanthin concentrations and changes in measures of physical activity. To determine the effects of the treatment and time of measurement on the measured variables, repeated measures analysis of variance and co-variance were used. Baseline cholesterol levels were used as a covariate as lutein is fat soluble and could be sequestered by cholesterol.

## 3. Results

Thirty-nine participants completed the study (Lutein *n* = 19, Placebo *n* = 20; 17 males, 22 females, Age: 67.5 ± 6.6 year, BMI 25.4 ± 2.6 kg/m^2^). Five participants withdrew from the study for personal reasons. There were no significant gender x treatment x time interactions (*p* > 0.29) so genders were pooled for analysis. Compliance with consuming the capsules was high and not different between treatments (Lutein 95.8 ± 4.1%, Placebo 95.1 ± 6.4%; *p* = 0.6).

There was no difference in baseline plasma lutein and zeaxanthin levels, cholesterol levels, average activity counts and steps per day, time spent undertaking sedentary, light physical activity and moderate to vigorous activity and exercise self-efficacy between treatments groups (*p* > 0.1, Table 1). Participants allocated to the lutein treatment weighed slightly less than those allocated to the placebo treatment at baseline. 

**Table 1:** Baseline characteristics for participants randomized to consume lutein or placebo capsules with milk.

Characteristic	Lutein	Placebo
Age (years)	67.7 ± 7.7	67.4 ± 5.5
BMI (kg/m^2^)	24.9 ± 2.5	25.8 ± 2.6
Weight (kg) **	70.3 ± 12.5	73.1 ± 10.6
Plasma lutein (μg/dL)	10.3 ± 2.5	10.1 ± 3.6
Plasma zeaxanthin (μg/dL)	4.3 ± 0.7	4.5 ± 0.7
Plasma lutein + zeaxanthin (μg/dL)	14.6 ± 2.9	14.6 ± 4.1
Cholesterol (mmol/L)	5.0 ± 0.8	4.8 ± 0.9
Accelerometer counts per day	235,292 ± 82,693	273,760 ± 85,018
Sedentary time (min/day)	235 ± 61	219 ± 46
Light physical activity (min/day)	301 ± 88	341 ± 76
Moderate to vigorous physical activity (min/day)	22 ± 14	24 ± 18
Steps per day	7685 ± 2234	7632 ± 2092
Exercise self-efficacy	1204 ± 345	1073 ± 425

Values are mean ± SD; BMI, body mass index; ** significantly different value between treatment groups at baseline (*p* < 0.001).

**Figure 3:**
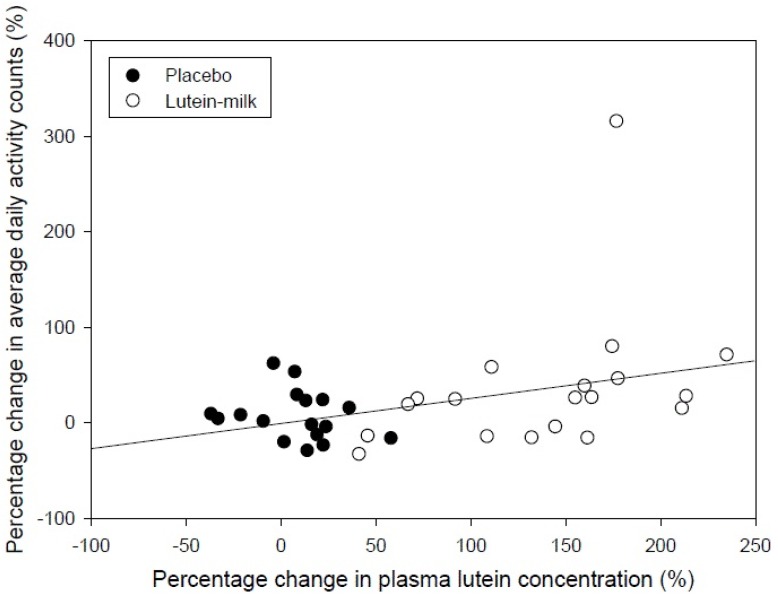
Relationship between percentage change in plasma lutein concentration and percentage change in average activity counts determined using accelerometry.

Lutein significantly increased plasma lutein and zeaxanthin concentrations during the study, and there was no change in the placebo group (*p* < 0.001, treatment × time; Table 2). The absolute change in plasma lutein concentration was not significantly associated with the absolute change in average daily activity counts (*r* = 0.29, *p* = 0.08). However, when expressed as a percentage, the percentage change in plasma lutein concentration was positively associated with the percentage change in average activity counts (*r* = 0.36, *p* = 0.03), such that participants who experienced a greater percentage increase in plasma lutein concentration had a greater increase in activity counts (Figure 3). Thus a doubling of plasma lutein concentration was associated with a 26% increase in average daily activity counts. One participant had a greater than 300% increase in average daily activity counts; excluding this person’s data from the correlation analysis did not alter the relationship between the percentage change in plasma lutein concentration and the percentage change in average daily activity counts. Changes in plasma zeaxanthin concentrations were not associated with changes in activity counts when expressed as either absolute values (*r* = 0.20, *p* = 0.25), or as percentage change (*r* = 0.18, *p* = 0.30).

**Table 2:** Change in values following 4 weeks of supplementation and 3 weeks of physical activity participation.

Outcome	Lutein	Placebo
Weight (kg)	0.04 ± 1.12	0.02 ± 0.66
Plasma lutein (μg/dL) **	13.9 ± 5.7	0.09 ± 2.5
Plasma zeaxanthin (μg/dL) *	0.58 ± 0.50	0.09 ± 0.88
Plasma lutein + zeaxanthin (μg/dL) **	14.5 ± 5.9	0.2 ± 2.7
Counts per day	49,995 ± 77,384	8528 ± 75,691
Sedentary time (min/day)	−3.4 ± 41.6	17.7 ± 42.8
Light physical activity (min/day)	19.0 ± 46.9	−14.2 ± 70.8
Moderate to vigorous physical activity (min/day)	8.6 ± 15.2	7.1 ± 17.2
Steps per day	1652 ± 1602	948 ± 2054
Exercise self-efficacy	43 ± 160	87 ± 230

Values are mean ± SD Significant time by treatment effect ** *p* < 0.001, * *p* = 0.04.

**Figure 4:**
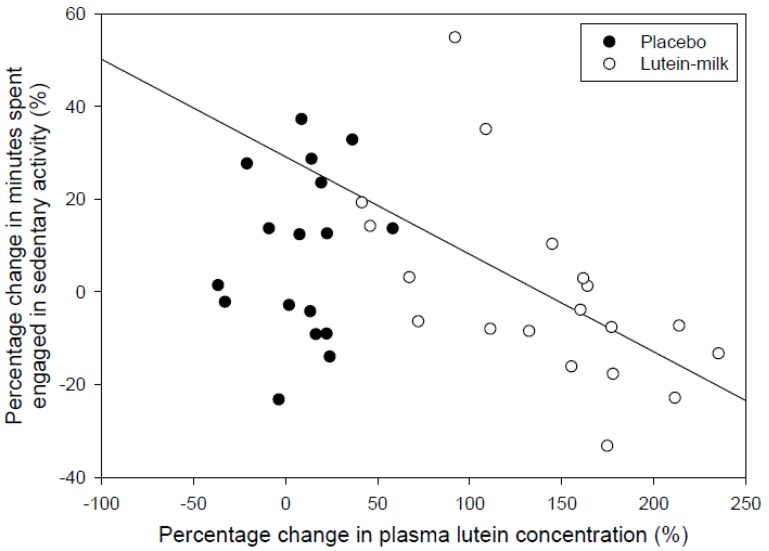
Relationship between percentage change in plasma lutein concentration and percentage change in minutes spent engaged in sedentary activity determined using accelerometry.

The change in plasma lutein concentration was inversely related to the change in the time per day (in minutes) that was spent being sedentary (*r* = −0.36, *p* = 0.03), such that those who experienced greater increases in plasma lutein concentrations experienced greater reductions in the time spent engaged in sedentary activities. For every 1 μg/dL increase in plasma lutein concentration there was on average a 1.9 min decrease in time spent being sedentary. This relationship was strengthened slightly when expressed as percentage change in time spent in sedentary activity relative to percentage change in plasma lutein (*r* = −0.39, *p* = 0.02), whereby a doubling of plasma lutein concentration was associated with a 10% decrease in time spent being sedentary (Figure 4). There was a trend for an inverse relationship between the change in plasma zeaxanthin concentration and time spent in sedentary activity (absolute change, *r* = −0.30, *p* = 0.07; percentage change, *r* = −0.32, *p* = 0.06).

Average activity counts increased by the end of the study period (*p* = 0.03), but with no difference in the increase between treatments (*p* = 0.11, *p* = 0.08 when controlling for cholesterol). The number of steps taken per day increased over the study period (*p* < 0.001), but with no difference between treatment groups (*p* = 0.24). There was no difference in self-reported minutes spent engaged in exercise between treatments during the three weeks of the exercise program (Lutein, 41.1 ± 21.3 min/day; Placebo, 32.0 ± 18.6 min/day; *p* = 0.16). Body weight and exercise self-efficacy did not change during the study (*p* = 0.94 and *p* = 0.16, respectively). Time spent engaged in light physical activity did not change significantly during the intervention (time, *p* = 0.81; time × treatment, *p* = 0.10). Time spent engaging in moderate to vigorous activity increased during the intervention period (*p* = 0.006), but with no difference in the magnitude of increase between treatments (*p* = 0.78). Sedentary time did not change significantly during the intervention (time, *p* = 0.32; time × treatment, *p* = 0.14). There was no difference between treatments in the other outcomes when controlling for cholesterol.

## 4. Discussion

The main finding of the present study was that increases in plasma lutein concentration correlated positively with changes in daily activity and inversely with changes in sedentary time, such that individuals who achieved the greatest increases in plasma lutein concentrations achieved the greatest increases in average daily activity counts and decreases in time spent engaged in sedentary activities. These changes may be important, given that low physical activity and increased time engaged in sedentary activity are associated with increases in cardio-metabolic disease risk (e.g., type 2 diabetes) [13] and mortality [14], particularly since supplementation with lutein increased average plasma lutein concentration by 135%, and a doubling of plasma lutein was on average associated with a 26% increase in activity counts and a 10% reduction in sedentary time. The average plasma lutein and zeaxanthin concentrations at baseline were similar to those reported in other studies [7,15,16,17,18].

The average daily activity counts recorded in this study were similar to those reported previously in older adults using accelerometry [19]. While there was an overall tendency for a greater increase in most measures of physical activity and a greater reduction in sedentary time in the participants who consumed lutein compared with those who consumed the placebo, these differences did not reach statistical significance (*p* = 0.08). Given the statistically significant relationship between the increase in plasma lutein concentrations and increases in physical activity and decreases in sedentary time, it seems that the high variance in the increase in physical activity and reduction in sedentary time overall may have been related to the high variability in increases in plasma lutein. There is evidence that lutein absorption varies considerably depending on the food matrix, and is particularly influenced by the amount of fat consumed at the same time, with higher fat intake promoting greater lutein bioavailability [20,21]. Thus, while the supplements were consumed with full-cream milk in an effort to increase absorption of the lutein, there was still considerable variability in the bioavailability, possibly in part due to the relatively low fat content of the milk and the influence of other foods consumed around the same time as the supplements. Strategies to increase the bioavailability of lutein might therefore promote increases in physical activity and reductions in sedentary time.

While this study provided evidence that lutein-milk supplementation can increase physical activity levels and reduce sedentary time, with the magnitude of change being associated with the magnitude of increase in plasma lutein concentration, the mechanism by which lutein elicits these effects is not clear. Lutein is able to cross the blood-brain barrier [22] and therefore has the potential to alter neural processes within the brain. Indeed, lutein consumption has previously been shown to be associated with cognitive performance in older women [23] and, as a result, we hypothesised that lutein might alter cognitions related to exercise behaviour and thus potentially increase engagement in physical activity. Exercise self-efficacy reflects a person’s belief that they will be able to engage in exercise, and has been shown in previous studies to be related to physical activity levels in older adults [19]. However, there was no change in exercise self-efficacy scores during the study period and there was no relationship between changes in exercise self-efficacy scores and changes in physical activity, indicating that in this study, the relationship between plasma lutein concentration and changes in physical activity and/or sedentary time were not mediated by changes in exercise self-efficacy. Future studies should seek to evaluate the mechanism by which an increase in plasma lutein concentration may increase physical activity and reduce sedentary time.

## 5. Conclusions

The current study provides preliminary evidence that the consumption of lutein increases plasma lutein concentrations, and that this increase is associated with increases in activity and reductions in time spent engaged in sedentary activities. Further evaluation of the potential for lutein to increase physical activity and reduce sedentary activity in older adults should be undertaken in larger, longer-term studies which evaluate the potential health benefits associated with these changes in physical activity and sedentary behaviours.
